# Histone methyltransferase G9a is a key regulator of the starvation-induced behaviors in *Drosophila melanogaster*

**DOI:** 10.1038/s41598-017-15344-2

**Published:** 2017-11-07

**Authors:** Kouhei Shimaji, Ryo Tanaka, Toru Maeda, Mamiko Ozaki, Hideki Yoshida, Yasuyuki Ohkawa, Tetsuya Sato, Mikita Suyama, Masamitsu Yamaguchi

**Affiliations:** 1 Department of Applied Biology, Kyoto Institute of Technology, Matsugasaki, Sakyo-ku, Kyoto, 606-8585 Japan; 2 The Center for Advanced Insect Research Promotion, Kyoto Institute of Technology, Matsugasaki, Sakyo-ku, Kyoto, 606-8585 Japan; 3 Department of Biology, Graduate School of Science, Kobe University, Nada, Kobe, 657-8501 Japan; 4 Department of Advanced Medical Initiatives, Faculty of Medicine, Kyushu University, Maidashi, Fukuoka, 812-8582 Japan; 5 Division of Bioinformatics, Medical Institute of Bioregulation, Kyushu University, Maidashi, Fukuoka, 812-8582 Japan

## Abstract

Organisms have developed behavioral strategies to defend themselves from starvation stress. Despite of their importance in nature, the underlying mechanisms have been poorly understood. Here, we show that *Drosophila* G9a (dG9a), one of the histone H3 Lys 9-specific histone methyltransferases, functions as a key regulator for the starvation-induced behaviors. RNA-sequencing analyses utilizing *dG9a* null mutant flies revealed that the expression of some genes relating to gustatory perception are regulated by dG9a under starvation conditions. Reverse transcription quantitative-PCR analyses showed that the expression of gustatory receptor genes for sensing sugar are up-regulated in starved *dG9a* null mutant. Consistent with this, proboscis extension reflex tests indicated that *dG9a* depletion increased the sensitivity to sucrose under starvation conditions. Furthermore, the locomotion activity was promoted in starved *dG9a* null mutant. We also found that *dG9a* depletion down-regulates the expression of *insulin-like peptide* genes that are required for the suppression of starvation-induced hyperactivity. Furthermore, refeeding of wild type flies after starvation conditions restores the hyperactivity and increased sensitivity to sucrose as well as *dG9a* expression level. These data suggest that dG9a functions as a key regulator for the decision of behavioral strategies under starvation conditions.

## Introduction

Behavioral epigenetics has attracted attentions for the last decade, because epigenetic regulation can induce rapid and long-lasting effects on gene expression in response to environmental changes. Although a numerous number of studies have revealed the biological roles of epigenetic factors over the last 40 years, how epigenetic regulation affects behaviors of organisms and how behaviors affect epigenetic regulation have just started to be investigated^1^. Previous studies reported that several epigenetic factors are relevant to behaviors included in learning/memory, neurodevelopmental disorders, drug addiction, parenting and stress responses in mammals^1^. Some of these relationships have also been found in *Drosophila melanogaster*, the model organism extensively used for genetic studies because of its short life span and the high homology to human genes^2^. For example, some *Drosophila* epigenetic factors like *Ash1* and *Suppressor of variegation 4–20 homolog 1* are associated with autism spectrum disorders, one of the neurodevelopmental disorders characterized by the impaired communication, restricted interests and hyperactivity^3^. Especially it is revealed that the *dG9a*, a *Drosophila* homolog of mammalian *G9a* ^4^ functions as the key regulator of learning and memory through alteration of histone modification^5^.

G9a has been identified in mammals as one of the histone H3 Lysine 9 (H3K9) specific methyltransferases (HMTases) that catalyzes both H3K9 mono-methylation and H3K9 di-methylation^6,7^. G9a has various biological roles including DNA replication^8^, genome imprinting^9,10^, developmental reprogramming^11^ and substance addiction^12^. Especially, G9a plays a critical role in embryogenesis, since *G9a* knockout mice show embryonic lethality in early stages due to severe growth defects^7,13^. Generally, G9a functions to suppress expression of its target genes through H3K9 methylation, although from some reports it may act as a co-activator to positively regulate some genes such as targets of the hormone-activated glucocorticoid receptor^14,15^. However, studies on *in vivo* functions of G9a utilizing the mouse model have not advanced efficiently because of the embryonic lethality of the G9a knockout mice. On the other hand, *dG9a* depletion exerted no effect on fly viability at all^16^. Therefore, *Drosophila melanogaster* is suitable for the functional analyses of G9a in adults. dG9a can catalyze the methylation of H3K9 in euchromatin regions and the methylated H3K9 contributes to heterochromatin formation and transcriptional repression of specific genes *in vivo* ^5,17^. Although the *dG9a* depleted flies show no viability defect^16^, previous reports utilizing *dG9a* knockdown flies or knockout flies revealed that dG9a has a regulatory role in the developmental process of germ cell line like spermatogenesis and oocyte specification^18,19^ as well as in learning and memory^5^. In contrast to the mammalian G9a, dG9a was not essential for *Drosophila* viability. Because of this unexpected finding, one can consider that the epigenetic regulation through H3K9 methylation is not developed to be critical for *Drosophila* viability and therefore dG9a plays no important role for *Drosophila* viability under laboratory conditions. However, it was important to note that while there are various environmental stresses in the wild, *Drosophila* was always maintained under optimal conditions in the laboratory. Therefore, we focused on the analyses under stressed conditions and recently revealed that dG9a has a critical role in acquisition of tolerance under starvation stress in adult stage through regulating the activity of autophagy^20^.

In terms of behavioral changes under starvation stress, *Drosophila* developed two behavioral strategies to increase the possibility that they can find new food sources^21,22^. Firstly, they increase responsiveness for a sugar taste by means of up-regulating the expression of *Gustatory receptor* (*Gr*) *64a*, a well-known gustatory receptor for sensing sugar^23^. Secondary, starvation stress induces hyperactivity through activating octopaminergic neurons whose functions can be regulated by glucagon and insulin signals^24^. However, it remains totally unknown whether there is a key regulator for the decision of these starvation-induced behaviors in spite of its importance in nature.

In this study, RNA-sequencing (RNA-seq) analyses followed by gene ontology (GO) analyses revealed that the expression of genes encoding gustatory receptors and odorant binding proteins are altered in *dG9a* mutants under starvation conditions. Further genetic analyses revealed that *dG9a* depletion increases the expression levels of gustatory receptor genes for sensing sucrose. Behavioral analyses revealed that *dG9a* depletion up-regulates the sucrose sensitivity in response to starvation stress. Our data suggest that dG9a regulates the starvation-induced shift of locomotion activity through controlling the expression of *insulin-like peptide* (*Ilp*) genes that are required for the suppression of starvation-induced hyperactivity. Refeeding of wild type flies after starvation conditions restored the hyperactivity and increased sensitivity to sucrose as well as *dG9a* expression level. Our data suggest that dG9a functions as a key regulator for the decision of behavioral strategies under starvation conditions.

## Results

### RNA-seq analyses of *dG9a*-depleted *Drosophila* under starvation conditions

**Figure 1:**
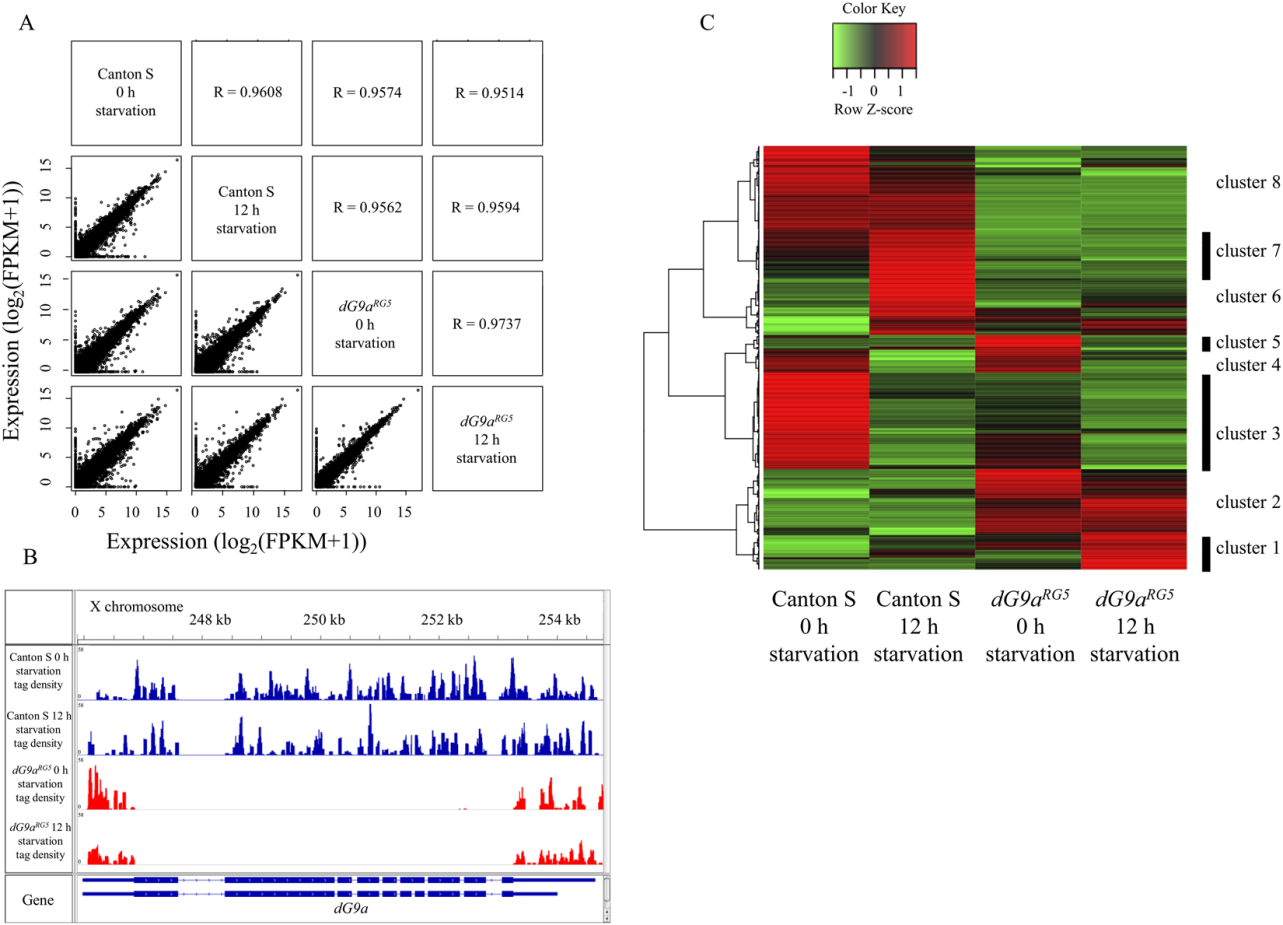
RNA-seq analyses utilizing starved *dG9a* depleted adult flies. (**A**) RNA-seq data using 0 h and 12 h starved adult flies of the *dG9a* null mutant (*dG9a* ^*RG5*^) and the wild type (Canton S). We added 1 to the FPKM value before log2 transformation to avoid a negative value and used this transformed value for the gene expression level. Horizontal axis represents the gene expression level in the group shown above the graph. Vertical axis represents the gene expression level in the group shown in the right of the graph. R value means the Pearson’s correlation coefficient between the group shown in below and in left. (**B**) RNA-seq analysis of the *dG9a* gene region. The x-axis represents genomic location on X chromosome. *dG9a* open reading frame is shown as the blue line in the ‘gene’ column. The y-axis represents tag density in Canton S (Blue) and *dG9a* ^*RG5*^ (Red) (**C**) Heatmap of differentially expressed genes. Eight clusters were generated based on the expression changes between Canton S and *dG9a* ^*RG5*^ as well as starvation conditions.

Previous study utilizing *dG9a* null mutant (*dG9a* ^*RG5*^) showed that dG9a plays an important role for the survival of adult fly under starvation stress conditions^20^. We confirmed that there is no difference in the life span of *dG9a* ^*RG5*^ mutant compared to that of Canton S under fed conditions (Fig S1). These results demonstrate that *dG9a* depletion affects fly viability under starvation conditions, but not under fed conditions. To determine which genes are regulated by dG9a under starvation stress, we performed RNA-seq analyses using adult flies of the *dG9a* null mutant and the wild type after 0 h and 12 h starvation. Thus, we made 4 groups; 0 h starved Canton S, 12 h starved Canton S, 0 h starved *dG9a* ^*RG5*^ and 12 h starved *dG9a* ^*RG5*^. Isolated RNAs were sequenced with a deep sequencer (Illumina HiSeq; Illumina, San Diego, CA, USA), which yielded an average of 40,508,694 1 × 51 bp single end reads per sample. These reads were aligned to the *Drosophila melanogaster* reference genome (UCSC dm6). The expression of in total 404 genes were differentially expressed between Canton S and *dG9a* null mutant across the analyses (*q-*value < 0.05). The expression levels of all detected genes were shown as fragments per kilobase of transcript per million mapped fragments (FPKM) in all combination of the groups (Fig. 1A). We calculated Pearson’s correlation coefficient between these groups (Fig. 1A). High correlation was detected in any combination of the groups (R > 0.95). The *dG9a* null mutant lacks the whole *dG9a* open reading frame (ORF)^16^. *dG9a* expression almost completely disappeared with almost no detectable reads mapped on the *dG9a* ORF in the *dG9a* null mutant (Fig. 1B). Among the 404 differentially expressed genes, we generated 8 clusters according to the type of expression changes and created a heatmap with two-dimensional hierarchical cluster analysis (Fig. 1C).

To identify biological features of differentially expressed genes, we carried out a GO analysis. The enriched biological process terms were analyzed with the Database for Annotation, Visualization and Integrated Discovery (DAVID; http://david.abcc.ncifcrf.gov) tool in the context of the GO classification with biological process (BP) terms. We focused on the cluster 1, 2, 6, 7 and 8, because genes included in these clusters are differentially expressed between 12 h starved *dG9a* null mutant and 12 h starved wild type (Fig. 1C). The all enriched BP terms with differentially expressed genes included in these clusters are shown in Table 1. The most enriched term was ‘innate immune response’ (*P* = 7.8 × 10^−13^) and the second was ‘response to bacterium’ (Table 1). These results are consistent with the previous study utilizing mouse model that revealed that G9a is recruited by the transcription factor ATF7 and regulates the expression of a group to genes involved in innate immunity in macrophages^25^.

**Table 1:** GO analysis of differentially expressed genes in starved *dG9a* ^*RG5*^.

Category	Term	P-Value	Benjamini
GOTERM_BP_DIRECT	innate immune response	7.80E-13	1.50E-10
GOTERM_BP_DIRECT	response to bacterium	6.30E-12	6.10E-10
GOTERM_BP_DIRECT	antibacterial humoral response	1.10E-09	6.80E-08
GOTERM_BP_DIRECT	defense response to Gram-positive bacterium	6.20E-07	3.00E-05
GOTERM_BP_DIRECT	cellular response to heat	2.30E-05	8.80E-04
GOTERM_BP_DIRECT	defense response	2.80E-05	9.00E-04
GOTERM_BP_DIRECT	defense response to bacterium	2.20E-04	5.90E-03
GOTERM_BP_DIRECT	response to heat	4.90E-04	1.20E-02
GOTERM_BP_DIRECT	multicellular organism reproduction	9.50E-04	2.00E-02
GOTERM_BP_DIRECT	cellular response to UV	2.80E-03	5.20E-02
GOTERM_BP_DIRECT	humoral immune response	3.10E-03	5.20E-02
GOTERM_BP_DIRECT	oxidation-reduction process	3.30E-03	5.20E-02
GOTERM_BP_DIRECT	response to oxidative stress	4.60E-03	6.60E-02
GOTERM_BP_DIRECT	proteolysis	5.00E-03	6.70E-02
GOTERM_BP_DIRECT	defense response to Gram-negative bacterium	5.30E-03	6.50E-02
GOTERM_BP_DIRECT	carbohydrate metabolic process	8.70E-03	9.90E-02
GOTERM_BP_DIRECT	response to UV	1.30E-02	1.40E-01
GOTERM_BP_DIRECT	nitrogen compound metabolic process	1.50E-02	1.50E-01
GOTERM_BP_DIRECT	defense response to fungus	2.00E-02	1.80E-01
GOTERM_BP_DIRECT	cellular response to unfolded protein	3.50E-02	2.90E-01
GOTERM_BP_DIRECT	defense response to protozoan	3.50E-02	2.90E-01
GOTERM_BP_DIRECT	hexose metabolic process	5.80E-02	4.20E-01
GOTERM_BP_DIRECT	wing disc development	8.80E-02	5.50E-01
GOTERM_BP_DIRECT	sensory perception of chemical stimulus	9.70E-02	5.70E-01

### *dG9a* null mutant shows the increased expression of gustatory receptor genes under starvation conditions

Our GO analyses detected the term ‘sensory perception of chemical stimulus’ (*P* = 9.7 × 10^−2^). *dG9a* depletion significantly changed the expression of several *Odorant binding proteins* (*Obps*) and *Gustatory receptors* (*Grs*) under starved conditions. The RNA-seq results of these genes, *Gr93d*, *Obp46a*, *Obp56a*, *Obp56b*, *Obp56e* and *Obp56f*, as well as other *Obps* and *Grs* are summarized in Table S1. We confirmed that the expression of *Obp56a*, *Obp56e*, *Obp56f* and *Gr93d* is significantly changed by *dG9a* depletion by the reverse transcription–quantitative PCR (RT-qPCR) analyses (Fig. S2). A number of *Drosophila* OBPs are expressed both in gustatory sensilla and olfactory sensilla^26^. Furthermore, some OBPs have been reported to be required for taste sensitivity^27–29^. Although the detailed functions of most of the differentially expressed *Obp* and *Gr* genes have not been clarified yet, we considered that *dG9a* might be responsible for regulating the expression of genes relating to gustatory senses. To address this point, we performed RT-qPCR analyses to examine the expression levels of the 8 gustatory receptor genes, that are well known to be required for the sense of sweet taste^30^, in 4 time points after starvation (0, 6, 12, 24 h). Among these genes, *Gr64b* and *Gr64c* are translated from the same transcript according to Flybase (http://Flybase.org). *Gr64d* and *Gr64e* are also translated from the same transcript.

**Figure 2:**
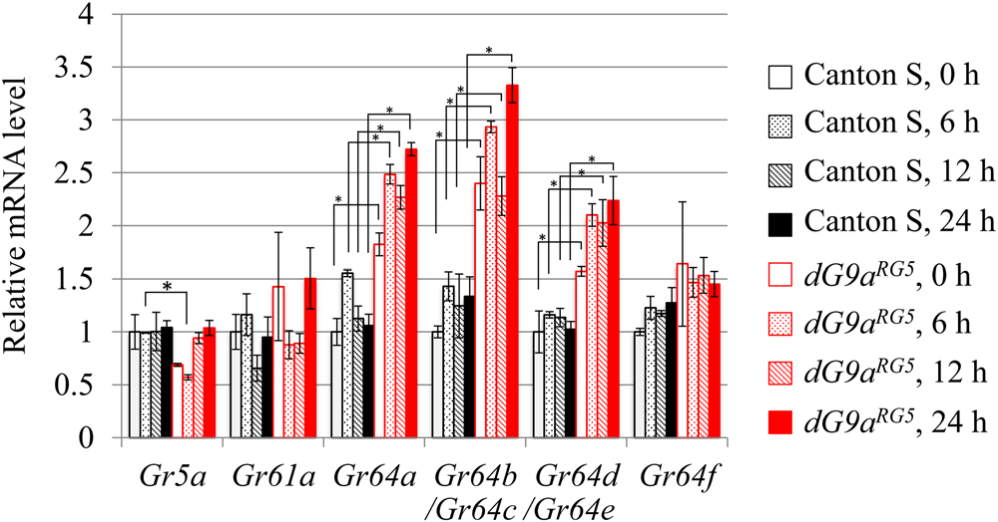
dG9a regulates the expression of gustatory receptor genes. Quantification of mRNA levels by RT-qPCR analyses of *Gr5a*, *Gr61a*, *Gr64a*, *Gr64b/c*, *Gr64d/e* and *Gr64f* in Canton S and *dG9a* ^*RG5*^ adult flies after 0 h, 6 h, 12 h and 24 h starvation. According to Flybase (http://Flybase.org), Gr64b and Gr64c are translated from the same transcript shown as *Gr64b*/*Gr64c*. Gr64d and Gr64e are also translated from the same transcript shown as *Gr64d/Gr64e*. Results were normalized to *β-tubulin* and are displayed as relative values for that of 0 h starved Canton S. n = 3. Error bars represent standard errors. *There is significant difference between Canton S and *dG9a* ^*RG5*^ in each time point evaluated by unpaired two-tailed Student t-tests. *P < 0.05. There was also significant difference in *Gr64a*, *Gr64b/c*, *Gr64d/e* between Canton S and *dG9a* ^*RG5*^ when a two-way factorial analysis of variance was applied (*P* < 0.05).

The RT-qPCR analyses indicated that *dG9a* depletion significantly up-regulated the expression of *Gr64a*, *Gr64b/Gr64c* and *Gr64d/Gr64e* under starvation conditions as well as fed conditions (*P* < 0.05), while it showed little effects on the expression of *Gr5a*, *Gr61a* and *Gr64f* (Fig. 2). *Gr5a* is reported to be necessary for sensing trehalose^31–33^. *Gr61a* is functional for sensing trehalose and glucose^34^. *Gr64f* is required for sugar detection in combination with other gustatory receptors^35^. *Gr64a-e* are also reported to sense sugar taste, especially *Gr64a* plays a critical role in sensing sucrose, maltose and glucose^35^. These results indicate that *dG9a* is responsible for suppressing the expression levels of the genes encoding gustatory receptors for sensing sweet taste. Although dG9a modifies the expression of a wide variety of genes through its epigenetic regulation as shown in RNA-seq analyses, the results of our RT-qPCR analyses led us to further examine the relationship between dG9a and the sense of taste under starvation conditions in the present study.

### *dG9a* null mutant shows increased sensitivity to sucrose under starvation conditions

**Figure 3:**
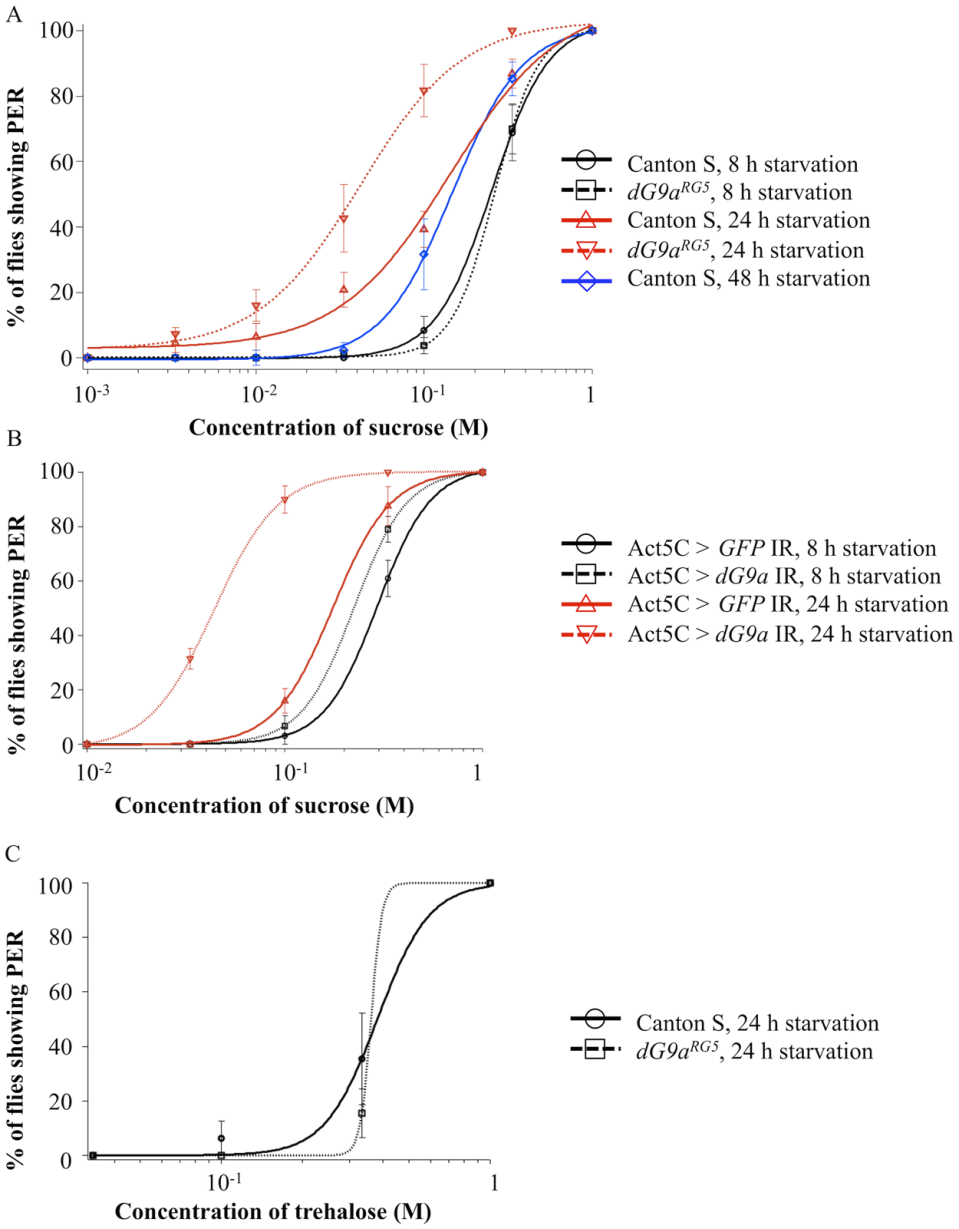
*dG9a* depletion increases the taste sensitivity to sucrose under starvation. (**A**) Sucrose concentration-PER curves in Canton S and the *dG9a* ^*RG5*^ under different starvation conditions (n = 5). (**B**) Sucrose concentration-PER curves in the *dG9a* knockdown fly (+; *GAL4* ^*Act5C*.*PI*^/*UAS-dG9a IR*;+) and control fly (+; *GAL4* ^*Act5C*.*PI*^/*GFP* ^*dsRNA*. *R*. *Scer/UAS*^;+) under different starvation conditions (n = 5). The *UAS-dG9a IR* strain was created previously (strain number 79)^17^. (**C**) Trehalose concentration-PER curves in the wild type and the *dG9a* null mutant under 24 h starvation conditions (n = 4). These sucrose concentration-PER curves are created by fitting the average of the results in each time point to Hill equation using Igor pro software (WaveMetrics). Error bars represent standard errors.

We then examined the sensitivity to sweet tastes in starved wild type and *dG9a* null mutant by proboscis extension reflex (PER) tests (Fig. 3A), one of the widely used behavioral assays for evaluating gustatory sensitivity in insects including *Drosophila* ^23,36^. When an attractive substance like sugar contacts to the chemosensilla, the fly extends its proboscis to feed it. Therefore, the taste sensitivity to specific substances can be evaluated by measuring the threshold changes of PER responses^23,36^. The *Gr64a* has been reported to be required for the detection of sugars including sucrose^35,37^, therefore we first examined PER responses to sucrose. The threshold of PER responses to sucrose of 24 h starved wild type flies (threshold: 0.13 ± 0.023 M (average ± standard error)) was significantly decreased compared to that of 8 h starved wild type flies (threshold: 0.25 ± 0.035 M) (*P* < 0.05). Such up-regulation of the sensitivity in the wild type (Canton S) under starvation was previously observed^38^. We also performed PER tests with *dG9a* null mutant at the same time. *dG9a* null mutant showed no significant difference in the threshold level of PER responses to sucrose in 8 h starvation conditions (threshold: 0.29 ± 0.023 M) in compared to the wild type (*P* > 0.05). However, *dG9a* depletion significantly increased the sensitivity in 24 h starved conditions (threshold: 0.047 ± 0.012 M) (*P* < 0.05). The sensitivity is not further increased after 24 h starvation in the wild type because the threshold of 48 h starved wild type (threshold: 0.17 ± 0.044 M) is not significantly changed in compared with that of 24 h starved wild type (*P* > 0.05), indicating that the increase of the sucrose sensitivity in *dG9a* null mutant is not due to the acceleration of starvation conditions. These results indicate that *dG9a* depletion increases the sucrose sensitivity in response to starvation.

We further confirmed these results with *dG9a* knockdown flies of whole bodies (Act5C > *dG9a* IR) (Fig. 3B). The threshold of PER responses to sucrose in 24 h starved Act5C > *dG9a* IR flies (threshold: 0.044 ± 0.0036 M) was significantly decreased in compared with that in the control flies (Act5C > *GFP* IR) (threshold: 0.157 ± 0.029 M) (*P* < 0.05), while there was no decrease under 8 h starved conditions (Act5C > *GFP* IR threshold: 0.30 ± 0.031 M, Act5C > *dG9a* IR: 0.24 ± 0.040 M) (*P* > 0.05). These data confirmed that the increase of the sucrose sensitivity in *dG9a* null mutant under 24 h starvation conditions shown in Fig. 3A is not due to the possible background mutation of the *dG9a* null mutant.

It is noted that disruption of *Gr5a* decreases the sensitivity to trehalose^31–33^. *dG9a* null mutant showed no significant difference in the threshold level of PER responses to trehalose in 24 h starvation conditions (threshold: 0.46 ± 0.068 M) in compared to the wild type (threshold: 0.36 ± 0.084 M) (*P* > 0.05) (Fig. 3C). This is consistent with the results that *dG9a* depletion has little effects on regulating the expression of *Gr5a* (Fig. 2).

### *dG9a* null mutant exhibits the hyperactivity under starvation conditions

The PER tests suggest that *dG9a* depletion promotes the taste sensitivity to sucrose in response to starvation conditions. Starved flies also promote their locomotion activity to increase the possibility that they can find desirable food^21,22^. Therefore, we next examined whether dG9a regulates the locomotion activity under starvation conditions or not. The locomotion activity was measured by the frequency that flies cross the infrared beam in the midpoint of the tubes of the *Drosophila* Activity Monitoring (DAM) system (Trikinetics) as described previously^39^.

**Figure 4:**
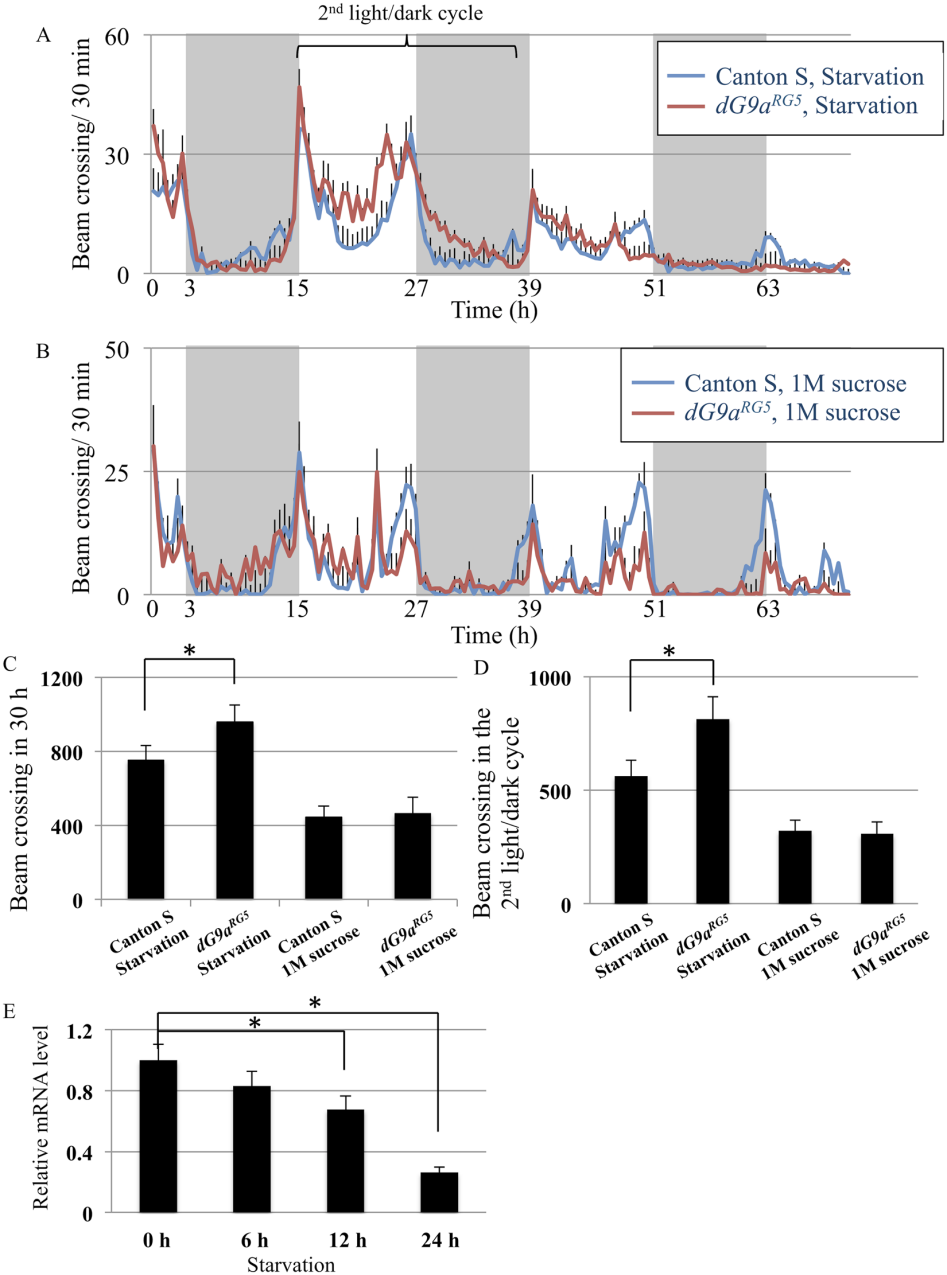
*dG9a* depletion increases the locomotion activity under starvation. (**A**) The beam crossing activity of Canton S and the *dG9a* ^*RG5*^ under starvation conditions. Gray and white background in the graph represents 12 h dark periods and 12 h light periods, respectively. (**B**) The beam crossing activity of Canton S and *dG9a* ^*RG5*^ in the presence of 1 M sucrose. (**C**,**D**) Averaged total crossing in 30 h after start point (**C**) and during 2^nd^ light/dark cycle (**D**). 0 h starved Canton S: n = 31. 0 h starved *dG9a* ^*RG5*^: n = 29. Canton S on 1 M sucrose: n = 24. *dG9a* ^*RG5*^ on 1 M sucrose: n = 20. *P < 0.05. (**E**) Quantification of mRNA levels by RT-qPCR analyses of *dG9a* in wild type adult flies after 0 h, 6 h, 12 h and 24 h starvation. Results were normalized to *β-tubulin* and are displayed as relative values for that of 0 h starvation (n = 3). Error bars represent standard errors. *P < 0.05.

We examined the locomotion activity of the wild type and *dG9a* null mutant under starvation conditions (Fig. 4A) and that with feeding 1 M sucrose solution as a control experiment (Fig. 4B). Interestingly the hyperactivity was observed with *dG9a* null mutant remarkably during the 2nd light/dark cycle under starvation conditions, whereas such hyperactivity was not occurred in the case with feeding 1 M sucrose solution (Fig. 4A,B). Since the *dG9a* null mutant starts to die after 30 h starvation^20^, we compared the total beam crossing during 30 h after the start point (Fig. 4C). The total beam crossing under starvation conditions in the *dG9a* null mutant exhibited 27% increase in compared with that in the wild type (Fig. 4C). Remarkably, the total beam crossing under starvation conditions in the *dG9a* null mutant exhibited 45% increase compared with that in the wild type during the 2nd light/dark cycle (Fig. 4D). These results indicate that *dG9a* is responsible for suppressing the locomotion activity under starvation and that this suppression is disrupted in the *dG9a* null mutant. Taken together with the results of PER tests (Fig. 3), we suggest that dG9a is functional for the suppression of both sucrose sensitivity and locomotion activity, which may result in longevity under starvation through saving energy. Consistent with our hypothesis, the expression of *dG9a* in adult whole body decreases gradually under starvation in wild type flies (Fig. 4D). The mechanisms of the starvation-induced regulation of locomotion activity and sucrose sensitivity may be too complex to be explained solely by *dG9a* expression in wild type flies, as starvation conditions may change a wide variety of genes. However, our results indicate the possibility that the hyperactivity and higher sucrose sensitivity observed in starved wild type flies occur by the lack of *dG9a*, as observed in the starved *dG9a* ^*RG5*^ mutant.

### *dG9a* depletion decreases the expression of *insulin-like peptides* under starvation conditions

**Figure 5:**
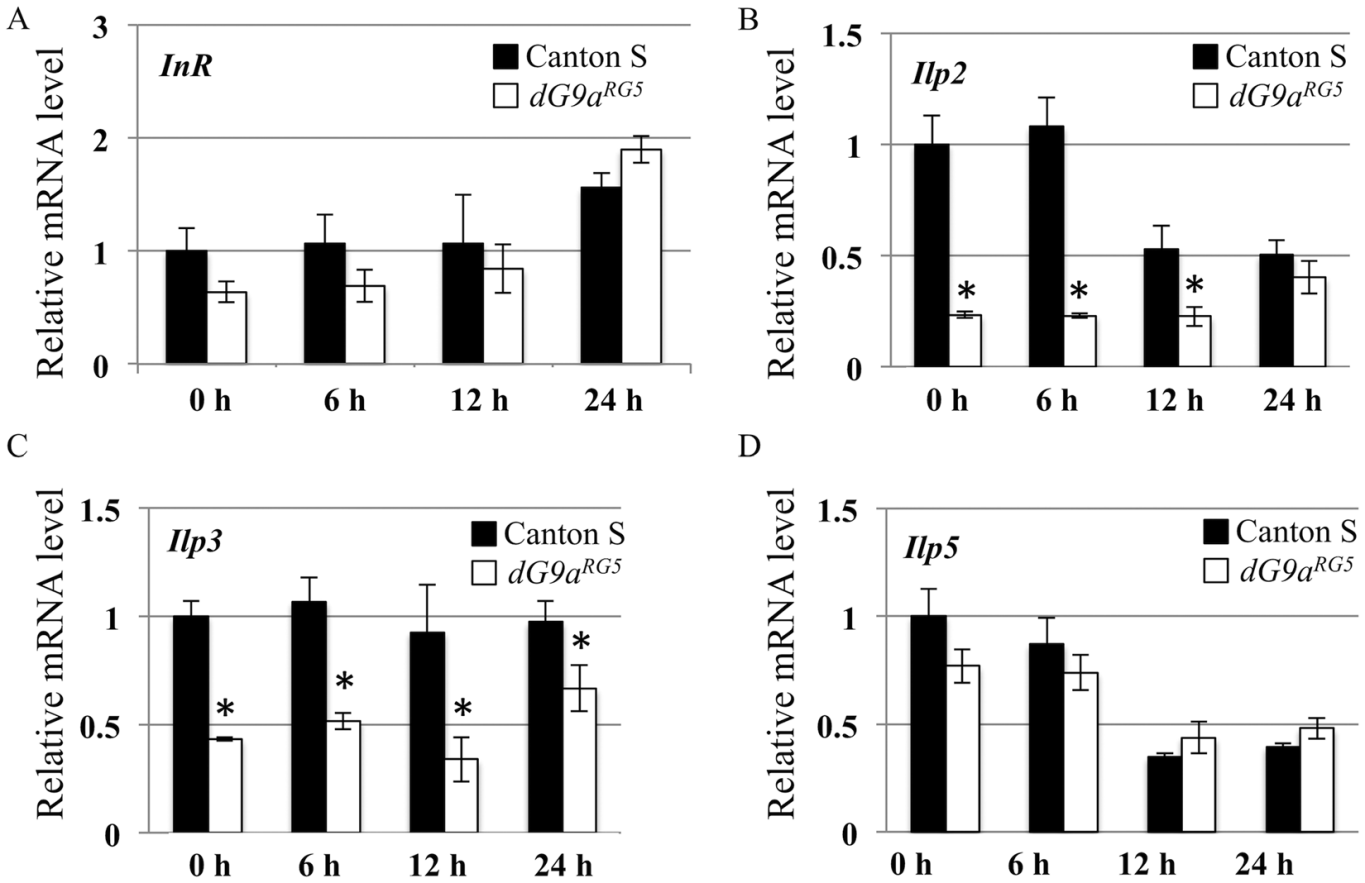
*dG9a* regulates the expression of *Ilp* genes. Quantification of mRNA levels by RT-qPCR analyses of *InR*. (**A**), *Ilp2* (**B**), *Ilp3* (**C**) and *Ilp5* (**D**) in Canton S and *dG9a* ^*RG5*^ adult flies after 0 h, 6 h, 12 h and 24 h starvation. Results were normalized to *β-tubulin* or *Gapdh1* and are displayed as relative values for that of 0 h starved Canton S (n = 3). Error bars represent standard errors. *P < 0.05.

The starvation-induced hyperactivity is caused by activating the octopaminergic neurons in *Drosophila melanogaster* ^24,38^. It also has been reported that the starvation-induced hyperactivity can be suppressed by Insulin-like peptides (Ilps) in *Drosophila* ^24^. Hence, we examined whether dG9a plays a role in regulating the expression of genes involved in these signals. Among the 7 *Ilp* genes (*Ilp1-7*) existing in the *Drosophila* genome, *Ilp2*, *3* and 5 are reported to be expressed in insulin producing cells during adult stage and the expression of these *Ilp*s decreases in response to starvation^40–42^. Interestingly, our RT-qPCR analyses showed that the *dG9a* depletion significantly reduced the expression levels of *Ilp*2 and *Ilp3* throughout the starvation, whereas it exerted no effect on the expression level of *Insulin-like receptor* (*InR*) and *Ilp5* (Fig. 5A–D). The hyperactivity was not induced in *dG9a* null mutant compared to wild type in fed conditions (Fig. 4B), although the expression levels of *Ilp2* and *Ilp3* were significantly reduced by *dG9a* depletion (Fig. 5C,D). Consistent with these observations, other studies reported that suppression of *Ilps* increases the locomotion activity under starvation conditions, but not under fed conditions^24^. There results suggest that *dG9a* is responsible for suppressing the locomotion activity under starvation stress by up-regulating the expression levels of *Ilp*s.

We found that *dG9a* regulates the expression of *Ilp* genes as well as *Gr64* genes (Figs 2 and 5). To examine whether *dG9a* regulates the expression of these genes directly or not, we performed Chromatin immunoprecipitation-quantitative PCR (ChIP-qPCR) assay with 0 h and 24 h starved flies. By this assay, we could not detect the dG9a binding to the genomic regions containing the promoters of *Ilp2*, *Ilp3*, *Ilp5*, *Gr64a* and *Gr64b/c* (Fig. S3), suggesting that dG9a regulates the expression of these genes rather indirectly at these time points. However, by the ChIP-qPCR assay, we cannot exclude the possibility that *dG9a* interacts only transiently with the target chromatin loci. Further detailed analyses will be required to reveal the mechanism how dG9a regulates the expression of these genes.

### Refeeding after fasting restores the hyperactivity and increased sensitivity to sucrose as well as *dG9a* expression

**Figure 6:**
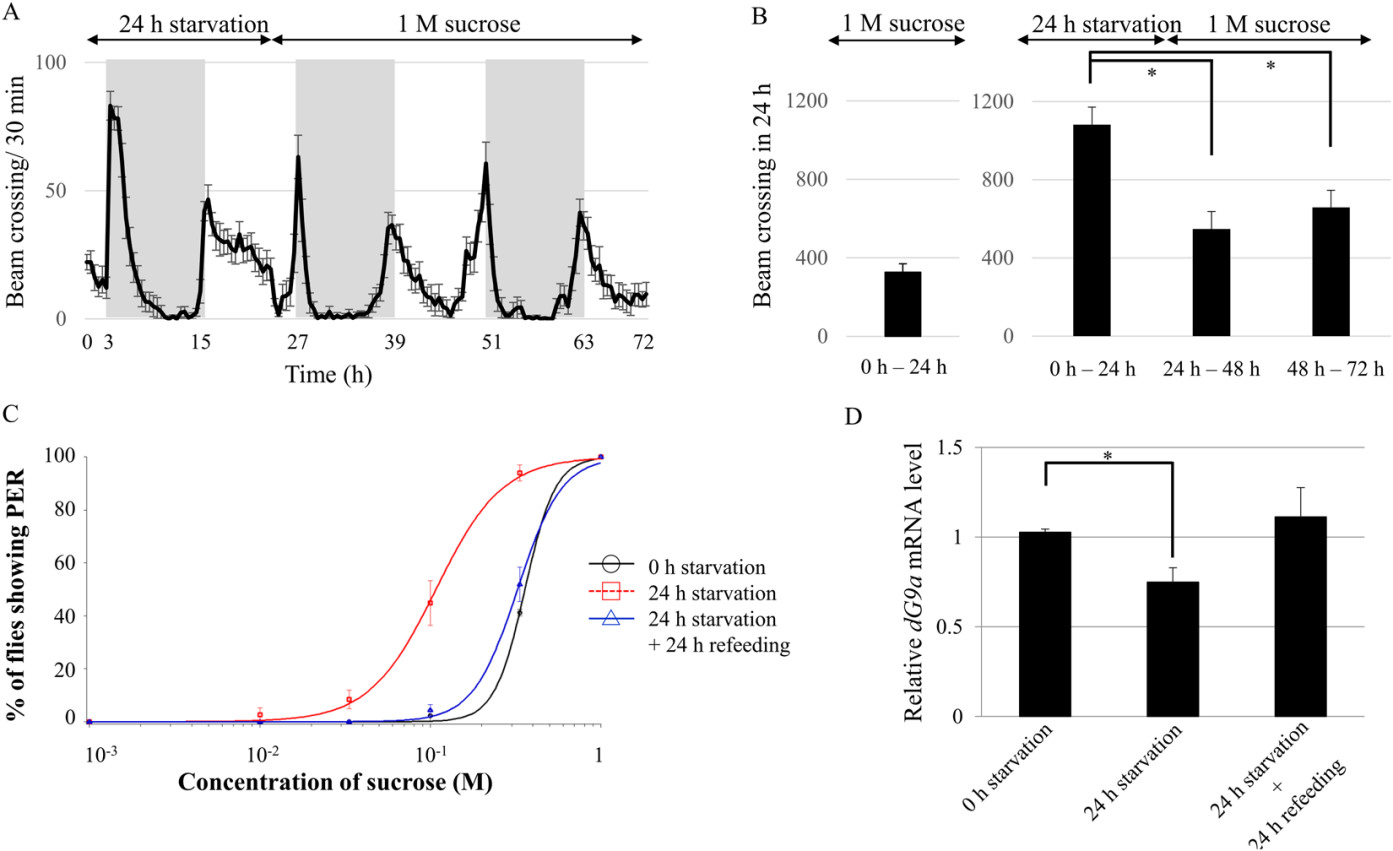
Refeeding after fasting restores the hyperactivity and increased sensitivity to sucrose as well as *dG9a* expression. (**A**) The beam crossing activity of Canton S under 24 h starvation conditions followed by 1 M sucrose refeeding. Gray and white background in the graph represents 12 h dark periods and 12 h light periods, respectively. (**B**) (left) Averaged total crossing in 24 h under 1 M sucrose feeding conditions. n = 24. (right) Averaged total crossing in 24 h under starvation conditions and refed conditions calculated from the data shown in Fig. 6A. n = 17. *P < 0.05. (**C**) Sucrose concentration-PER curves in Canton S under different starvation conditions and refed conditions (n = 3). (**D**) Quantification of mRNA levels by RT-qPCR analyses of *dG9a* in wild type adult flies after 0 h, 24 h starvation and after 24 h starvation followed by 24 h 1 M sucrose refeeding. Results were normalized to *β-tubulin* and are displayed as relative values for that of 0 h starvation (n = 3). Error bars represent standard errors. *P < 0.05.

We observed that starvation conditions induce hyperactivity (Fig. 4C) and increased sensitivity to sucrose (Fig. 3A) as well as decreased *dG9a* mRNA level (Fig. 4D). Next, we examined whether refeeding after starvation conditions restores these changes or not. Firstly, we examined the locomotion activity of the wild type under 24 h starvation followed by refeeding of 1 M sucrose (Fig. 6A). The total beam crossing during 24 h starvation conditions exhibited 70% increase in compared with that in 1 M sucrose fed conditions (Fig. 6B). The total beam crossing during 24 h refeeding after 24 h starvation conditions exhibited 49% decrease in compared with that in 24 h starvation conditions (Fig. 6B). Secondly, we examined the sensitivity to sucrose by PER tests. The threshold of PER responses of wild type flies under 24 h starvation conditions followed by 24 h refeeding of 1 M sucrose (threshold: 0.31 ± 0.018 M) was significantly increased compared to that of 24 h starved wild type flies (threshold: 0.11 ± 0.014 M) (*P* < 0.05) (Fig. 6C). There was no significant difference between PER thresholds of refed flies and that of 0 h starved flies (threshold: 0.34 ± 0.010 M) (*P* = 0.13) (Fig. 6C). Finally, we examined the mRNA level of *dG9a* by RT-qPCR. Remarkably, 24 h refeeding rescued the reduced mRNA level of 24 h starved flies to the similar level to that of 0 h starved flies (*P* = 0.31 between 0 h starved flies and refed flies) (Fig. 6D). These results indicate that refeeding after fasting restores the hyperactivity and increased sensitivity to sucrose as well as *dG9a* expression. Moreover, these results support the idea that the hyperactivity and higher sucrose sensitivity observed in starved wild type flies are dependent on the expression level of *dG9a* and that the regulation of behaviors by dG9a is highly responsive to the surrounding nutrient conditions.

## Discussion

In the present study, we found that *dG9a* depletion increases the sucrose sensitivity in response to starvation conditions. We also found that dG9a regulates the locomotion activity under starvation conditions by controlling the expression of *Ilp* genes.

Prior to this study, we found that *dG9a* null mutant flies are sensitive to starvation and explored the underlying mechanism. dG9a is functional for saving energy through recycling cellular components by regulating the expression of genes required for autophagy^20^. In addition to this process, we here further found that dG9a functions as a suppressor of starvation-induced hyperactivity. This is also preferable for saving energy under starvation conditions. In nature, animals are exposed to starvation frequently, however, foraging require the costs of food-seeking energy as well as the threats from predation and environmental changes along with their migration^43–45^. Therefore, this foraging strategy requires assumption that the nutrient-poor conditions do not last long. Moreover, the strategy appears to be not effective under the conditions that there is no food available near them. Another way to survive under starvation is saving energy without moving like the hibernation associated with seasonal fluctuations of food availability, which can be observed in a wide range of animals including *Drosophila* ^46,47^. Our data indicated that dG9a suppresses the starvation-induced hyperactivity and that wild type flies exhibit the hyperactivity along with reduction of *dG9a* expression (Fig. 4). These data suggest that the wild type flies save energy without moving at the early phase of starvation and they become active to seek foods with risks along with the reduction of *dG9a* expression at the late phase of starvation. Therefore, our data suggest that dG9a functions as a key regulator for flies to decide these strategies depending on the time course of starvation and has an adaptive advantage to survive starvation conditions.

We performed RNA-seq analyses to identify which genes are regulated by dG9a under starvation stress (Fig. 1). Our GO analyses indicated that the most enriched term was ‘innate immune response’ and the second was ‘response to bacterium’ (Table 1), which suggests that there is strong relationship between dG9a and innate immune responses. In *Drosophila*, starved conditions and the following disruption of insulin signaling induce the expression of 4 antimicrobial peptides (AMPs), *Metchnikowin* (*Mtk*), *Drosocin* (*Dro*), *Drosomycin* (*Drs*) and *Attacin-A* (*AttA*)^48^. Our RNA-seq analyses detected the significant increase in the expression of all of these 4 antimicrobial genes under 12 h starved *dG9a* null mutant (Table S1). We examined the expression of these representative antibacterial genes by RT-qPCR using Canton S and *dG9a* ^*RG5*^ kept under same conditions. The expression of 3 representative genes, *Mtk*, *Dro* and *Drs*, showed similar expression pattern to that observed in the results of RNA-seq analyses (Fig. S4). These results indicate that the expression pattern of antibacterial genes was not due to accidental infection of one set of the flies, but truly due to *dG9a* mutation. These observations suggest a link between activation of innate immune system and starvation. Previous reports suggested that the induction of AMPs may help maintaining and enhancing the defense activity in particular when organisms are exposed to poor conditions like starvation^48^. Our data indicates the possibility that *dG9a* null mutant acquires the excess defense activity to bacterial infection under starvation conditions.

We revealed that the *dG9a* depletion increases taste sensitivity to sucrose under 24 h starved conditions (Fig. 3). Our RT-qPCR results indicated that *dG9a* depletion significantly increased the expression levels of genes encoding gustatory receptors for sensing sugar taste under 24 h starved conditions (Fig. 2). These results suggest that the sucrose sensitivity under 24 h starved conditions is dependent on the expression levels of gustatory receptor genes regulated by dG9a. However, the *dG9a* depletion does not increase sucrose sensitivity under earlier (8 h) starved conditions (Fig. 3), although we detected the significant increases in gustatory receptor genes under 0 h and 6 h starved *dG9a* null mutant (Fig. 2). Moreover, none of these genes in wild type flies are up-regulated under 12 h and 24 h starvation conditions in compared to non-starved conditions (Fig. 2). These results suggest that the starvation-induced increase of the sucrose sensitivity in wild type flies as well as increase of the sucrose sensitivity under non-starved conditions are dependent on underlying mechanisms other than expression changes of gustatory receptor genes, including the alterations of translation levels of *Gr* mRNAs, localization of *Gr* mRNA/proteins and the responses of higher order gustatory circuits in the brain like the feeding behavior control by neuropeptides like dRYamides^49–51^. Further analyses are required to clarify these mechanisms.

## Materials and Methods

### Fly stocks

Fly stocks were cultured at 25 °C on standard food. Canton S was used as the wild-type strain. The *dG9a* ^*RG5*^ flies were kindly provided by Dr. P. Spierer and used as the *dG9a* null mutant^52^. The *dG9a* ^*RG5*^ flies show no defects in viability compared to Canton S in 1st instar larvae, pupae and adult stages^52^. The *dG9a* ^*RG5*^ flies were back-crossed 10 times with Canton S to adjust the genetic background to Canton S. These backcrossed flies showed decreased viability under starvation conditions with similar extent of those with heterozygous mutant (*dG9a* ^*RG5*^ */dG9a* ^*del34*^) and Fat-body specific knockdown of *dG9a* by RNAi^20^. The *UAS-FLAG-IR dG9a* (strain #79) fly stock was produced previously^17^ and used as the *dG9a IR* flies in this paper. The y^*1*^ *w* ^*^; *GAL4* ^*Act5C*.*PI*^ */CyO* flies were used as the *Act5C-GAL4* flies. The *w* ^*111*^; *GFP* ^*dsRNA*. *R*. *Scer/UAS*^ flies were used as the *GFP IR* flies. All other stocks used in this study were obtained from the *Drosophila* Genomics and Genetic Resources, Bloomington *Drosophila* Stock Center and Vienna *Drosophila* RNAi Center.

### Life span assay

In the life span assay, newly merged adult flies were placed into vials on standard food at 25 °C. Every 3 days, they were transferred to new vials containing fresh standard food and the number of living flies was monitored until all had died. All assays were performed under non-crowded conditions (<20 flies per vial) and the results from 5 independent assays were combined. Graphs were generated with the Kaplan-Meier method by GraphPad Prism 6 software. Significance was calculated with Log-rank tests using GraphPad Prism 6 software.

### RNA-seq analysis

Five days old adult flies were placed into vials including a piece of paper soaked with 1.0 mL PBS under non-crowded conditions (20 flies per vial) and were collected after 0 h and 12 h with each fly line. Thus, we made 4 groups; 0 h starved Canton S, 12 h starved Canton S, 0 h starved *dG9a* ^*RG5*^ and 12 h starved *dG9a* ^*RG5*^. RNAs were isolated using Trizol^®^ reagent (Invitrogen) from 10 flies. The extraction procedures were repeated twice and samples were combined in each group for the analysis. Isolated RNAs were used for comparison with Illumina mRNA-seq libraries using a TruSeq Stranded mRNA Sample Prep Kits (Illumina) according to the manufacturer’s instructions. The obtained libraries were subjected to sequencing on a HiSeq. 1500 system (Illumina) for 51 cycles. We aligned the mRNA-seq reads to the reference *Drosophila melanogaster* genome sequence (UCSC dm6) using the TopHat^53^ program version 2.1.0 with the option ‘–library-type fr-secondstrand’. Cufflinks^54^ (version 2.2.1) was then employed with the option ‘-u–library-type fr-secondstrand’ to assemble transcripts and to calculate FPKM. Differentially expressed genes were also identified using Cuffdiff in the Cufflinks program package. The datasets generated during our RNA-seq analyses are available in the NCBIs Gene Expression Omnibus (GEO, https://www.ncbi.nlm.nih.gov/geo/) and are accessible through GEO Series accession number GSE93144.

### Cluster analysis

Two-dimensional hierarchical cluster analysis of differentially expressed genes was performed to generate a heatmap. The distance between every pair of genes was calculated as 1.0 - (Pearson’s correlation coefficient in FPKM between the 2 genes). The Ward’s linkage algorithm was used for cluster analysis. The heatmap was created using the heatmap.2 function of the ‘gplots’ packages in R language (https://www.r-project.org).

### Gene ontology analysis of differentially expressed genes

To gain biological insights into differentially expressed genes, we analyzed genes that were significantly altered with q-values of <0.05. For this the Gene Ontology (GO) classification system was applied, employing the database for annotation, visualization and integrated discovery, DAVID version 6.8^55^.

### Reverse transcription–quantitative PCR (RT-qPCR)

Three replicates of 20 starved adult male flies were collected at each starvation periods. The starvation conditions were the same to those written in the RNA-seq analysis. Total RNA was isolated using Trizol^®^ reagent (Invitrogen) from whole body and cDNA was synthesized using PrimeScript RT reagent kit (TaKaRa) according to the manufacturer’s instructions. Samples were run in duplicates with SYBR^®^ Premix Ex Taq^TM^ II (TaKaRa) using CFX96 touch^TM^ (Biorad) and the data were analyzed with standard curve-based method calculated with CFX Manager^TM^ software. Specificity of primers was tested with melt curves created by CFX Manager^TM^ software and agarose gel electrophoresis of amplified fragments. *β-tubulin* and *Gapdh1* were used as internal controls. Primer sequences of examined genes are listed below:

*β-tubulin* forward 5′-GAGACGTACTGCATCGACAAC-3′

*β-tubulin* reverse 5′-CAGGGAGACAAGATGGTTCAG-3′

*Gapdh1* forward 5′-GGAGCCACCTATGACGAAATC-3′

*Gapdh1* reverse 5′-TCGAACACAGACGAATGGG-3′

*Gr5a* forward 5′-CGCACTCTTTATCGCTCCATAG-3′

*Gr5a* reverse 5′-GCAGGCAGATGAAGTACAGATT-3′

*Gr61a* forward 5′-GAGGGTCTGAATGCCAAGAATA-3′

*Gr61a* reverse 5′-GAACTCCAGGGCAGCATTAT-3′

*Gr64a* forward 5′-ATGTTCACCCACCTGCTAAA-3′

*Gr64a* reverse 5′-AGGCGAATGAAGACCACATAG-3′

*Gr64b/c* forward 5′-GCAGCAAATGCCGAATGAA-3′

*Gr64b/c* reverse 5′-ATAGCACGATCAAACCGGATAA-3′

*Gr64d/e* forward 5′-CGCCTGGATGGTGTTCTTTA-3′

*Gr64d/e* reverse 5′-CAAGCCTCGACACATGAGAA-3′

*Gr64f* forward 5′-CTGGTCTTGATAGTGGCTCTTAAT-3′

*Gr64f* reverse 5′-TGGTCAACTGTGTCTTGTACTG-3′

*dG9a* forward 5′-TCAGATGGCCTATCTCCTTC-3′

*dG9a* reverse 5′-CAGTCCGCAGTTCATAATCC-3′

*Akh* forward 5′-CGTCCAGTGTCAATTGACCTTCTC-3′

*Akh* reverse 5′-AGCAGCATTTCGTTGGAGGTCTTG-3′

*InR* forward 5′-GGTACCAGAAGTCAGAGAACAAG-3′

*InR* reverse 5′-CGCTCCAAACTGTCGATAAGA-3′

*Ilp2* forward 5′-ACGAGGTGCTGAGTATGGTGTGCG-3′

*Ilp2* reverse 5′-CACTTCGCAGCGGTTCCGATATCG-3′

*Ilp3* forward 5′-GTCCAGGCCACCATGAAGTTGTGC-3′

*Ilp3* reverse 5′-CTTTCCAGCAGGGAACGGTCTTCG-3′

*Ilp5* forward 5′-ATGGACATGCTGAGGGTTG-3′

*Ilp5* reverse 5′-CGCCAAGTGGTCCTCATAAT-3′

*Mtk* forward 5′-GCAACTTAATCTTGGAGCGATTT-3′

*Mtk* reverse 5′-GGTCTTGGTTGGTTAGGATTGA-3′

*Dro* forward 5′-ATTCTGCCCGCCTAAAGATG-3′

*Dro* reverse 5′-TGCTGTCTTTCGTGTGTTTATTG-3′

*Drs* forward 5′-AAGTACTTGTTCGCCCTCTTC-3′

*Drs* reverse 5′-CACAGGGACCCTTGTATCTTC-3′

### Proboscis extension reflex (PER) test

The PER tests^23,36^ were performed using flies after 8 h or 24 h starvation for Fig. 3. We used 8 h starved flies for evaluating the PER responses during the earlier stage of starvation to reduce the variations in the data. Five days old flies were used for PER tests. To induce dG9a knockdown in whole body, *Act5C-GAL4* flies were crossed with *dG9a IR* flies and F1 progenies were used for tests. *Act5C-GAL4* flies were crossed with *GFP IR* flies and F1 progenies were used as a control. The starvation conditions were the same to those written in the RNA-seq analysis. Seven concentration steps of sucrose or trehalose solutions (1, 1/3, 1/10, 1/30, 1/100, 1/300 and 1/1000 M) were prepared with distilled water for stimulation. Each fly was fixed at the tip of the 200 μL yellow tip. They were satiated with distilled water prior to the PER tests. Labellar gustatory sensilla were stimulated with a droplet of sugar solution with a pipette tip under a binocular microscope. Ingestion of the sucrose solutions may affect the starvation conditions and PER. Therefore, we stimulated the sensilla by touching the tip of them with a droplet of sugar solution in a moment not to feed flies. 15–20 flies were tested with the concentration series of sucrose starting with the lowest concentration with >5-min interval to eliminate the adaptation effect of the last stimulation. PER thresholds were calculated by fitting the data to the Hill equation using Igor pro software (WaveMetrics). The PER thresholds were defined as the concentration of sucrose which induced PER in the half of the population. We repeated this PER tests 3–5 times and calculated the average threshold.

### Locomotion activity test

Fly strains were kept on 12 h light/dark cycles for at least 5 generations before performing locomotion activity tests. Individual 3–5 day-old adult male fly was placed in glass tube and its locomotion activity was continuously recorded by DAM2 *Drosophila* Activity Monitor (TriKinetics). One end of glass tubes was filled with cotton wool soaked with 0 M or 1 M sucrose solution and the other was sealed with PARAFILM® (Bemis). These instruments were put in the incubator at 25 °C and kept on 12 h light/dark cycles.

### Chromatin immunoprecipitation-quantitative PCR (ChIP-qPCR)

Fourty adult flies (3–5 days old) were collected after 0 h and 24 h starvation. The starvation conditions were the same to those written in the RNA-seq analysis. The flies were homogenized in a mortar after freezing with liquid N_2_ and DNA was cross-linked with 16% formaldehyde for 5 min. After cross-linking, we performed ChIP using ChIP Assay Kit (Millipore) according to the manufacturer’s instructions. In brief, cross-linked DNA was cut into fragments by sonication (Bioruptor, COSMO BIO) and immunoprecipitated with 0.2 μg of anti-dG9a antibody^17^ for 16 h. After washing and reverse cross-linking, DNA was isolated and performed qPCR with SYBR® Premix Ex Taq^TM^ II (TaKaRa) using CFX96 touch^TM^ (Biorad). The data were analyzed with standard curve-based method. The primers were designed in the site of −600 to −100 bp from the transcriptional start site, because dG9a is reported to bind to these upstream regions in the target genes by ChIP-sequencing analyses performed utilizing larvae^5^. Primer sequences used in this study are listed below:

*Ilp2* forward 5′-GGCGCGGATTGGTAAGTATAG-3′

*Ilp2* reverse 5′-CCGTACTAAGCCCTGCATTTAT-3′

*Ilp3* forward 5′-CTCCTTGGGCGAGAAAGTAAA-3′

*Ilp3* reverse 5′-CTGGCATGGGATGTCTGAAA-3′

*Ilp5* forward 5′-TACGCTAATCCATGGGCATAAA-3′

*Ilp5* reverse 5′-GCAGGAGCGTACTTGCTAAT-3′

*Gr64a* forward 5′-ACATGTATCTCGGCAGCTAATC-3′

*Gr64a* reverse 5′-GACACTTCTGTGGGAATTGGA-3′

*Gr64b/c* forward 5′-GACTGCTCTTTGGAGTGAGTAA-3′

*Gr64b/c* reverse 5′-GACAATGGTTCCAGCCATCTA-3′

*MAGE* forward 5′-CAGACGAAGAACATGGCTACTT-3′

*MAGE* reverse 5′-GCGTTCCCGCTTCAGATATT-3′

### Refeeding assay

Five days old adult Canton S were placed into vials including a piece of paper soaked with 1.0 mL PBS under non-crowded conditions (20 flies per vial) for 24 h and subsequently into the vials including a piece of paper soaked with 1.0 mL 1 M sucrose for 24 h. We performed PER tests and RT-qPCR analyses utilizing flies collected in 24 h and 48 h after the start point. For locomotion activity tests, the wild type flies were put under starvation conditions for the first 24 h and then put under 1 M sucrose feeding conditions.

### Statistical analysis

Unpaired two-tailed Student t-tests were performed to evaluate the difference of the average thresholds in PER assay, and to analyze the results of locomotion analyses and qPCR analyses. Compared results were considered statistically significant when the *P*-value < 0.05.

## Electronic supplementary material

Supplementary Information

Table S1
