# Supplementary material for: Histone methyltransferase G9a is a key regulator of the starvation-induced behaviors in Drosophila melanogaster
Source: Sci Rep. 2017 Nov 7;7:14763. doi: 10.1038/s41598-017-15344-2 (PMC5676964; doi:10.1038/s41598-017-15344-2)
Supplement: Supplementary file 1 — Supplementary Information [file 41598_2017_15344_MOESM1_ESM.pdf]

Supplementary information

Histone methyltransferase G9a is a key regulator of the starvation-induced behaviors in *Drosophila melanogaster*

Kouhei Shimaji[1,2], Ryo Tanaka[1,2], Toru Maeda[3], Mamiko Ozaki[3], Hideki Yoshida[1,2], Yasuyuki Ohkawa[4], Tetsuya Sato[5], Mikita Suyama[5] and Masamitsu Yamaguchi[1,2*]

[1]Department of Applied Biology, [2]The Center for Advanced Insect Research Promotion, Kyoto Institute of Technology, Matsugasaki, Sakyo-ku, Kyoto, 606-8585, Japan

[3]Department of Biology, Graduate School of Science, Kobe University, Nada, Kobe, 657-8501, Japan

[4]Department of Advanced Medical Initiatives, Faculty of Medicine, [5]Division of Bioinformatics, Medical Institute of Bioregulation, Kyushu University, Maidashi, Fukuoka, 812-8582, Japan

**Correspondence*: Phone, +81 75 724 7781; Fax, +81 75 724 7799; E-mail, myamaguc@kit.ac.jp

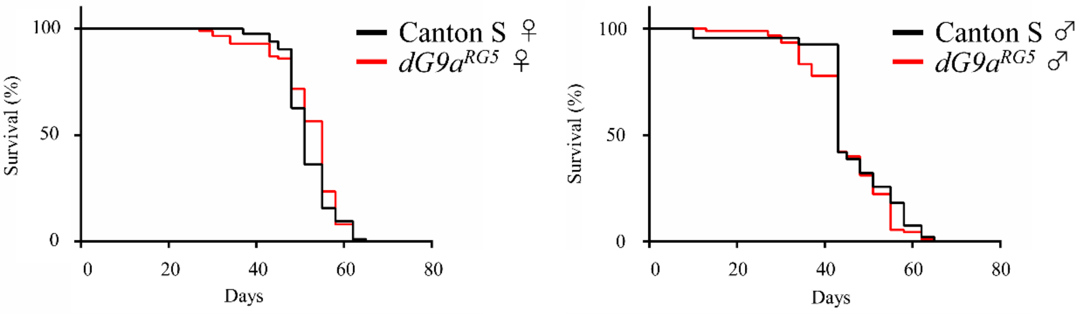

Figure S1 The results of life span assays using males and females of the wild-type (Canton S) (♀; n=83 from 5 independent experiments, ♂; n=93 from 5 independent experiments) and *dG9a* null mutant (*dG9a*^{RG5}) (♀; n=85 from 5 independent experiments, ♂; n=90 from 5 independent experiments). ♀; *P* = 0.18. ♂; *P* = 0.09.

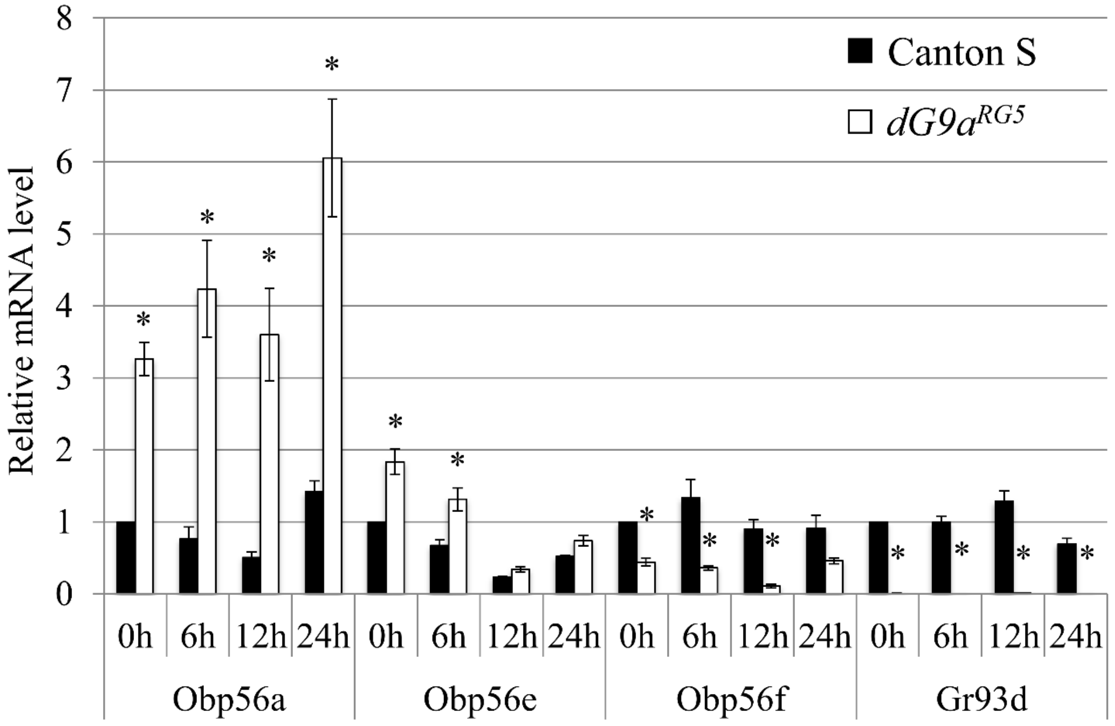

Figure S2 Quantification of mRNA levels by RT-qPCR analyses of *Obp56a, Obp56e, Obp56f* and *Gr93d* in Canton S and *dG9a*^{RG5} adult flies after 0 h, 6 h, 12 h and 24 h starvation. Error bars represent SE. n=3. * P <0.05.

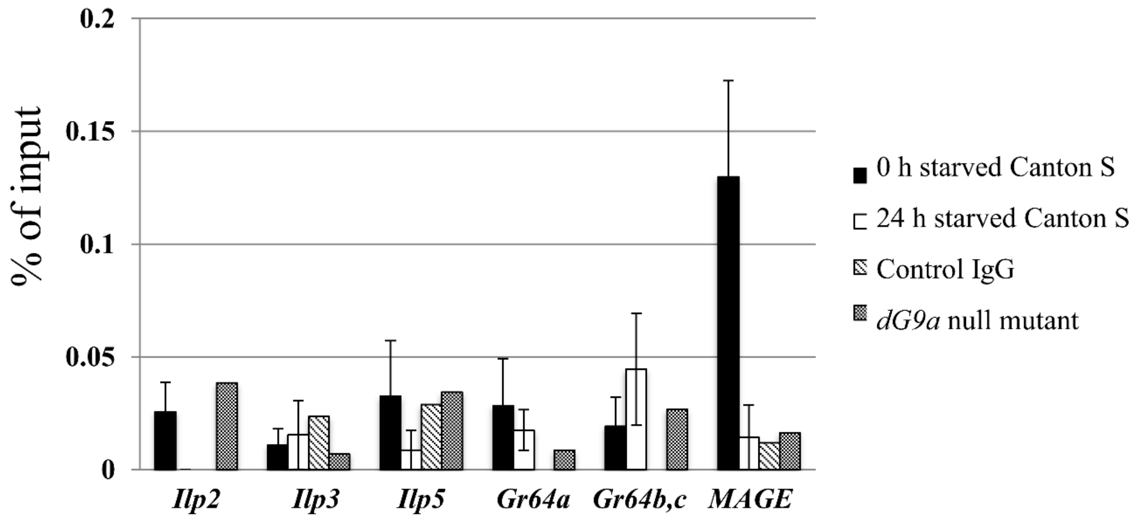

Figure S3 dG9a does not bind to the promoter region of *Ilp* genes and *Gr64* genes. (B) The results of ChIP assays using the anti-dG9a antibody as well as control IgG in Canton S and *dG9a*^{RG5}. Immunoprecipitated DNA was analyzed by RT-qPCR with primers for *Ilp2, Ilp3, Ilp5, Gr64a, Gr64b/c* and *Drosophila MAGE*. Results were normalized to inputs and shown as %. MAGE is a well-known direct target of G9a in mouse (Tachibana 2006) and *Drosophila* (Shimaji 2015). Canton S: n=3. Control IgG, *dG9a* null mutant: n=1.

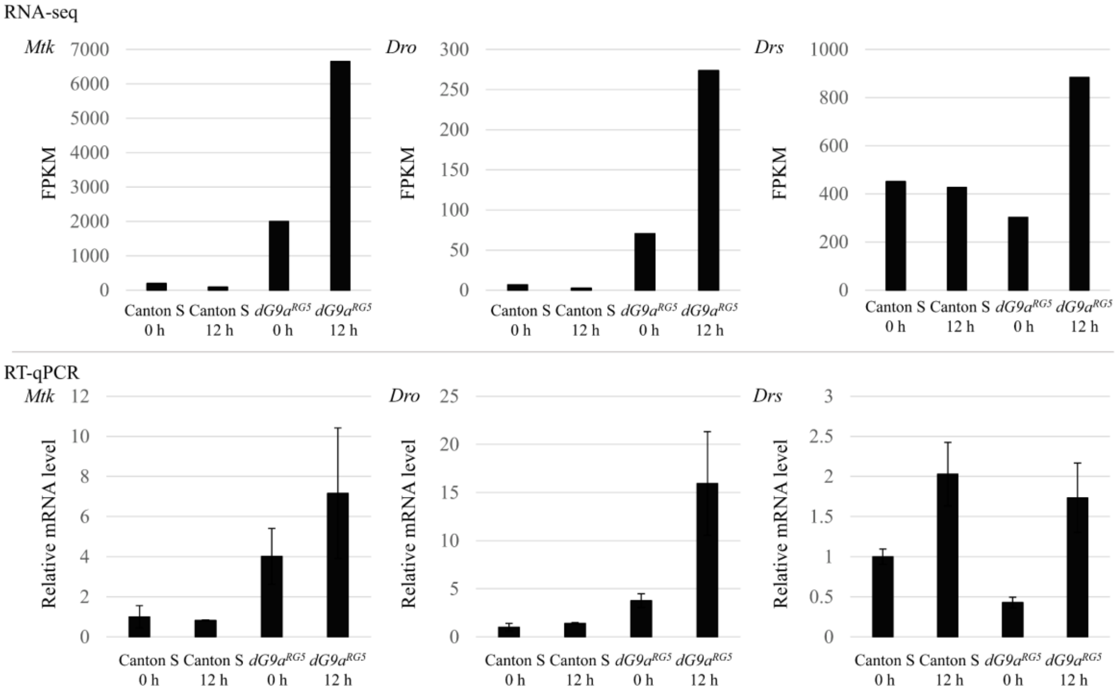

Figure S4 Quantification of mRNA levels by RNA-seq analyses (upper) and RT-qPCR analyses (bottom) of *Mtk, Dro* and *Drs* in Canton S and *dG9a*^{RG5} adult flies after 0 h and 12 h starvation. Error bars represent SE. RT-qPCR: n=3.
